# Gun Free Zones in Alcohol-Serving Establishments and Risk for Firearm Violence: A Cross-Sectional, Geospatial Study in Texas

**DOI:** 10.1007/s11524-024-00928-x

**Published:** 2024-11-06

**Authors:** Paul M. Reeping, Hannah S. Laqueur, Rose M. C. Kagawa

**Affiliations:** 1 Department of Emergency Medicine, UC Davis Violence Prevention Research Program, UC Davis, Sacramento, CA USA; 2 California Firearm Violence Research Center, Sacramento, CA USA

**Keywords:** Gun violence, Gun violence prevention, Gun-free zones, Epidemiology, Alcohol, Violence

## Abstract

To date, there have been no peer-reviewed studies in the United States estimating the impact of gun-free zone policies in alcohol-serving establishments on rates of firearm violence in and around such establishments. In this study, we utilized a cross-sectional design to estimate the impact of Texas’s 51% alcohol law, which prohibits the carrying of firearms in establishments that generate over half of their revenue from alcohol sales. The analysis focused on the difference in shooting incidents in and around establishments with and without firearm carrying prohibitions in 2021 and 2022. After adjusting for establishment type (bar/restaurant), alcohol sales volume, census tract level demographic factors, and the number of nearby restaurants and bars, results indicated that gun-prohibiting bars experienced significantly fewer shootings compared to those that allowed guns. Specifically, establishments that were gun-prohibited had 37% fewer shootings within 50 m than those that were gun-allowing, with a 95% confidence interval ranging from 60% fewer to 0.2% fewer. This association was more prominent in bars than in restaurants. The protective association with gun-prohibited status diminished with increased distance from the establishments; results were not significant at 100 m. Our study findings align with research suggesting that gun-free zones can reduce firearm violence. However, future studies using quasi-experimental designs that can better support causal inference are needed to support such a conclusion, as are studies exploring the efficacy of such policies in various settings and over longer periods.

## Introduction

In 2022, there were 48,117 fatalities due to firearm violence in the United States (U.S.), making firearm violence a leading cause of death [1]. Alcohol consumption is recognized as one of the most well-established individual-level risk factors for firearm injuries and fatalities. Among state prisoners, 34% report being under the influence of alcohol when they committed the violent offense for which they are serving time [2]. Toxicology studies find similar evidence for alcohol consumption surrounding criminal behavior, with one to two-thirds of perpetrators having elevated blood alcohol levels [3]. Additionally, a systematic review found that over one-third of firearm violence decedents had consumed alcohol before their death, and over a quarter had drunk heavily [4]. Given this strong link between alcohol consumption and firearm violence, it is not surprising that fifteen states have banned the concealed carry of firearms in bars and restaurants that serve alcohol [5].

Prohibitions on carrying firearms in establishments that sell alcohol are just one type of place-based firearm carrying restriction. Place-based restrictions, often known as gun-free zones, are premised on the idea that certain locations, due to their specific function or the vulnerability of their occupants, should be free from the presence of firearms to minimize the potential for violence or silencing speech [6]. Others have argued that rather than reducing violence, gun-free zones may increase firearm violence as they disarm only law-abiding citizens and deprive them of the opportunity for defensive gun use in the event of a violent encounter [7]. The logic behind this argument is that an individual who is legally carrying a firearm could potentially prevent (through deterrence), stop, or mitigate a violent situation, thus preventing or reducing the harm caused by would-be perpetrators. This perspective suggests that locations designated as gun-free zones effectively become “soft targets” for those intent on committing violence [8], and these critics assert that perpetrators may be more inclined to target these areas precisely because they know that occupants will not be armed and therefore will be less able to mount an effective defense. Even though the limited research on the topic is generally inconclusive [9], and one study of school zones suggests their gun-free status provides a small level of protection [10], many Americans believe that gun-free zones are less safe [11, 12].

The Supreme Court ruling in New York State Rifle & Pistol Association Inc. v. Bruen in 2022, which struck down New York’s law that restricted the carrying of firearms outside the home to only those who could demonstrate “proper cause,” has also brought place-based firearm policies into the spotlight [13]. While Bruen did not specifically address gun-free zones, its broader implications could affect them. With more limited ability to define *who* can carry firearms, some states have moved to define *where* firearms may be carried. However, these efforts have also met with significant litigation. The Bruen decision suggested that restrictions on carrying firearms must be justifiable within the historical context of the Second Amendment. Since this ruling, place-based litigation has increased from 4 to 17% of overall firearm-related litigation, [13] and successful challenges to place-based litigation increased from 18 to 69% [13].

Amid heightened scrutiny of place-based firearm regulations, and the known connection between alcohol use and increased risk of violence, research on the effects of banning firearms from places that sell alcohol is more urgent than ever. To the authors’ knowledge, this is the first study to examine the impact of prohibiting the concealed carrying of firearms in places that serve alcohol. Specifically, we examine Texas’s 51% alcohol law, which bans firearms in establishments deriving over half of their revenue from alcohol. These premises must display a specific sign (often referred to as a “51%” or “red” sign) indicating that carrying firearms is not permitted. We hypothesize that, after controlling for establishment type (bar/restaurant), alcohol sales volume, census level factors, and the number of nearby restaurants and bars, there will be fewer shootings in and around establishments designated as gun-free compared to those that are gun-allowing.

## Methods

### Data and Measures

Our sample includes establishments serving alcohol (bars and restaurants) and the area immediately surrounding them in Texas in 2021 and 2022. Bars and restaurants were geo-coded and mapped, and we cast buffers of 50 m from the address of each bar or restaurant. Our decision to use a 50-m (Euclidean) buffer was informed by several considerations. First, we aimed to capture firearm-related events occurring both within the establishment and in the immediate surrounding area, where violence is often more likely to occur. Incidents of alcohol-related violence often spill into the street or occur at entry and exit points. A smaller buffer risks missing these events. Additionally, we chose this buffer size in light of practices used in other gun-free zones, such as the 1000-foot perimeters often applied around schools, which are intended to regulate firearm presence in surrounding areas. In contrast, our 50-m buffer is relatively conservative, focusing on the direct vicinity of the establishment. As part of our robustness checks, we also tested a larger 100-m buffer to ensure that our results were not overly sensitive to the area of influence. Only those establishments for which we had complete data (described further below) were included in our primary analyses.

The outcome for this study was the location (latitude, longitude) of shootings that occurred in 2021 and 2022 in the 50-m buffer surrounding each bar and restaurant. If buffers overlapped and a shooting occurred within 50 m of more than one bar or restaurant, the shooting was assigned to the establishment to which it was closest. These data were obtained from the Gun Violence Archive, an online archive of gun violence incidents collected in real time from over 7500 law enforcement, media, government, and commercial sources [14]. Research comparing GVA data with city crime reports has validated the accuracy of these data since 2019 [15].

The primary exposure was the gun-free status of restaurants and bars in Texas in 2021 and 2022. Any establishment that received more than 51% of its revenue from alcohol is required by law to post a sign (colloquially known as a “red sign”) that declares the prohibition on the carrying of firearms on the premises. These data were obtained from the Texas Alcoholic Beverage Commission (TABC) Public Inquiry System [16]. The Texas Public Information Act (formerly known as the Texas Open Records Act) requires the government to make these records available upon request [17].

The total amount of alcohol sold by the establishment in 2021 and 2022 was obtained via the “Mixed Beverage Gross Receipts” data, via the Texas Open Data Portal [18]. This dataset included the total revenue from liquor, wine, and beer sold. The gun-free status and alcohol revenue variables were then linked using taxpayer number and location address. The primary units of study were those establishments for which we could link these data.

We included a number of additional hypothesized confounders as covariates in our models. Because the density of alcohol outlets and volume of alcohol consumption are related to the risk of violence in an area [19, 20], we included the amount of revenue from alcohol for each primary unit (bar or restaurant), the number of gun-prohibiting and gun-allowing restaurants and bars within 200 m of the primary units (as two separate covariates, and obtained from the Texas Alcoholic Beverage Commission (TABC) Public Inquiry System [16]), and the total revenue from the sale of alcohol in establishments within 200 m of the primary unit (obtained via the “Mixed Beverage Gross Receipts” data, via the Texas Open Data Portal [18]). We utilized a 200-m radius (rather than a 100-m radius) to include a relatively broad area in which there might be environmental and social influences on gun violence. The type of establishment (either bar or restaurant) was also obtained from the TABC Public Inquiry System [16] and included as a covariate as we hypothesized bars were more likely to meet the 51% rule and tend to draw different clientele and host different activities than do restaurants (e.g., outings with friends vs. family outings). We hypothesized that gun-prohibiting and gun-allowing establishments would differ in terms of sociodemographic factors related to violence risk. For example, areas with higher population density [21], more young adults, [22], and concentrated poverty [23] may face higher levels of gun violence. Additionally, firearm violence is more often located in highly segregated Black or Latino neighborhoods due to decades of systemic racism [24]. Higher-income individuals typically consume more alcohol [25], but factors such as segregation and disinvestment may also shape alcohol sales [26], and although off-premise alcohol outlets are more commonly found in communities of color [27], the evidence is mixed for on-premise outlets [28]. Therefore, we included census tract level measures of population density, the percentage of the population that is racialized as non-white the percentage of the population living in poverty, median income, and the percentage of the population aged 18–35 to account for these potential confounders. These variables were obtained from the U.S. Census Bureau American Community Survey (ACS) [29] for 2022.

### Statistical Analysis

To estimate the association between gun-free poster presence and the number of shootings within 50 m of each establishment, we employed a Generalized Estimating Equations (GEE) model with a quasi-Poisson distribution. This method accounts for the correlation of measurements taken from the same establishments across 2 consecutive years (2021 and 2022). We used an exchangeable correlation structure to handle the within-group correlation, which assumes a constant correlation between any two observations within the same group. Robust standard errors were calculated to provide valid inference under the quasi-Poisson assumption, which adjusts for overdispersion in the count data. A fixed effect for year was also included. Given that some of these establishments did not sell any alcohol in 2021 or 2022, we also conducted analyses excluding these units. Additionally, to mitigate the impact of outliers and ensure a focus on more typical cases, we trimmed the data to include only units within the 5th to 95th percentile range of alcohol sales in sensitivity analyses [30]. This approach excludes establishments at the extremes—both those with unusually low and unusually high alcohol sales—which might operate differently from the majority. Models were implemented separately for all establishments, bars only, and restaurants only. Finally, we conducted a sensitivity analyses using a 100 m buffer in place of the 50 m buffer, and in which we excluded data from January through March 10, 2021, as Texas did not fully reopen following COVID-19 shut-downs until this date [31].

## Results

### Descriptive Results

The number of restaurants and bars in 2021 and 2022 in Texas obtained from the TABC Public Inquiry System was 24,703. The number of restaurants and bars included in the Mixed Beverage Gross Receipts was 24,081. The total number of units that we were able to link was 11,118, of which 972 (8.7%) were red sign establishments (i.e., guns prohibited). Among the linked establishments, 8570 were restaurants, of which 196 (2.3%) were gun-prohibited, and 2548 were bars, of which 776 (30.5%) were gun-prohibited. Across all units and 2 years, the median revenue from alcohol was $272,070 per year, the mean was $499,934, the minimum was $0, and the maximum was $13,016,303. Among units that were gun-prohibited, the median revenue was $208,484 per year. Among units that were gun-allowing, the median revenue was $277,585 per year.

**Fig. 1:**
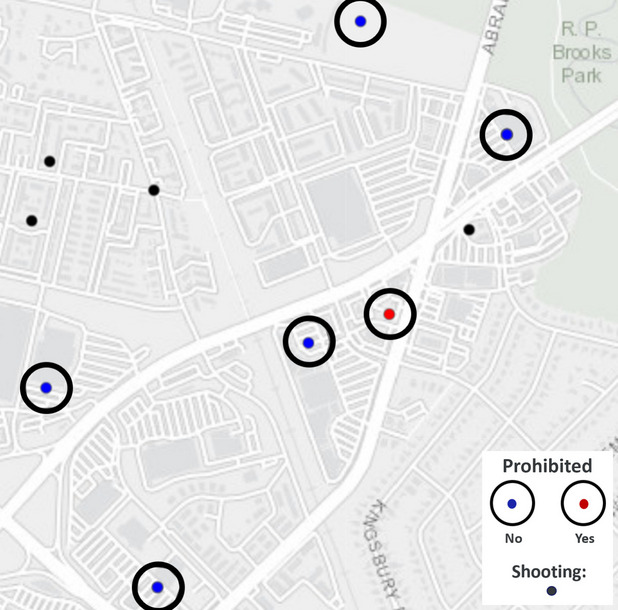
An example map outside of Dallas, Texas, showing the location of gun-prohibited/gun-allowing restaurants or bars, their associated 50-m buffers, and shootings

According to the Gun Violence Archive, the total number of shooting incidents in Texas in 2021 was 4462 and in 2022 was 3719. Figure 1 presents a map of an example area in Dallas, including the location of gun-prohibited/gun-allowing restaurants or bars, their associated 50-m buffers, and shootings.

A crosstabulation of the average number of shootings that occurred within 50 m of a unit, based on the gun-prohibited status of the unit and if the unit was a bar or a restaurant, is presented in Table 1. Gun-prohibited bars had a lower average number of shootings within 50 m than gun-allowing bars. On the other hand, gun-prohibited restaurants had a higher average number of shootings within 50 m than gun-allowing restaurants. These descriptive results have not been adjusted for potential confounders.

**Table 1:** The average number of shootings in 2021 and 2022 by gun-prohibited status and establishment type

	Restaurant	Bar	Both
Gun-prohibited	0.020	0.035	0.032
Gun-allowing	0.016	0.070	0.025
Both	0.018	0.053	0.026

**Fig. 2:**
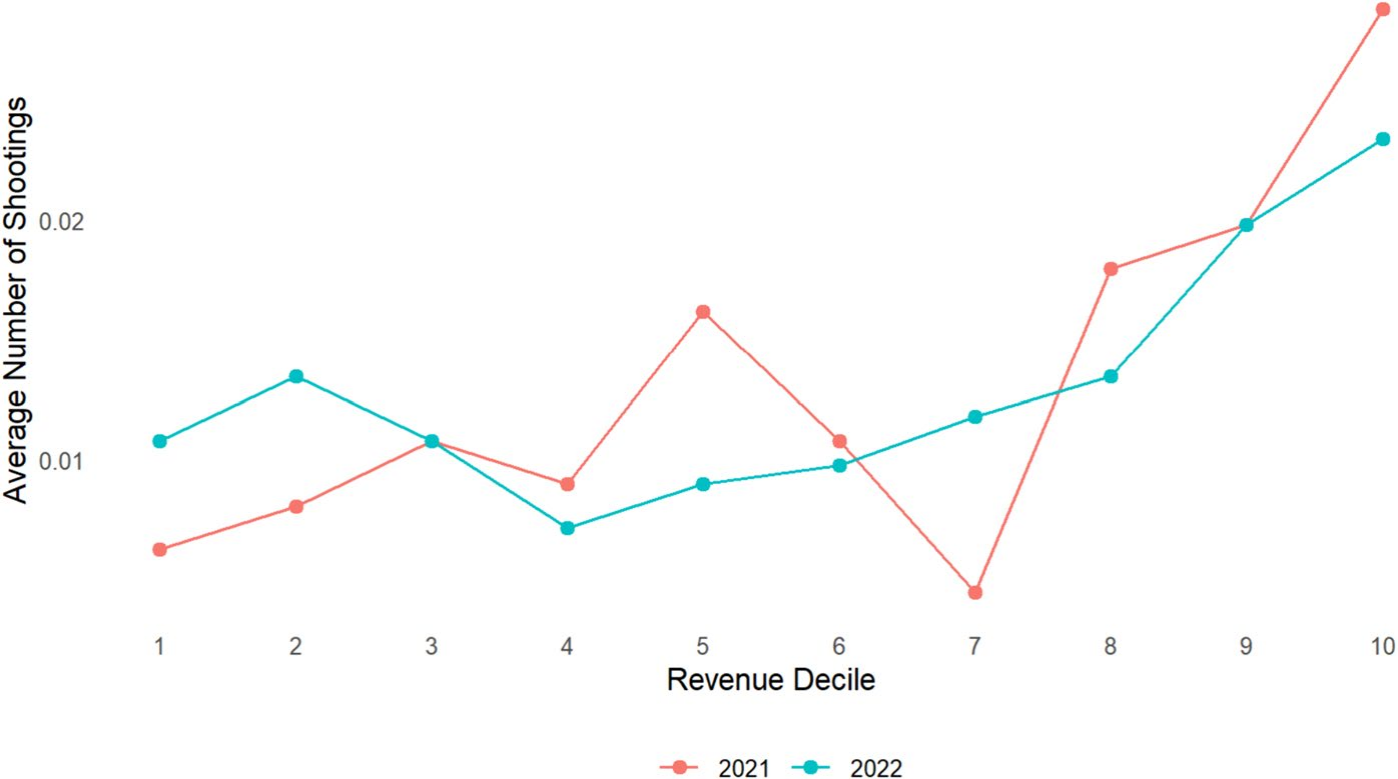
Average number of shootings within 50 m by decile of revenue from alcohol sales in bars and restaurants in Texas in 2021 and 2022

The average number of shootings within 50 m of an establishment by decile of revenue from alcohol sales in the establishments from 2021 and 2022 is illustrated in Fig. 2. While the first six deciles appear to not change meaningfully, there is a consistent increase in the number of shootings starting in the 7th decile to the 10th.

### Analytical Results

The adjusted rate ratio between gun-prohibited status of an establishment and the number of shootings within 50 m is given in Table 2. Overall, establishments that were gun-prohibited had 37% fewer shootings within 50 m than those that were gun-allowing, with a 95% confidence interval ranging from 60% fewer to 0.2% fewer. These results were consistent in the models excluding establishments without any alcohol sales and establishments with the bottom 5% and top 5% of revenue from alcohol sales excluded, although less powered. Bars that were gun-prohibiting had 40% fewer shootings within 50 m than those that were gun-allowing, with a 95% confidence interval ranging from 63% fewer to 3% fewer. For the restaurant-only models, the confidence intervals were wide, indicating a high level of uncertainty about the direction of the relationship. A table showing the associations between all of the covariates and the outcome is included in Appendix Table 3, [32].

**Table 2:** Adjusted rate ratios for shootings within 50 and 100 m of establishments based on gun-free status and alcohol sales revenue*

	Main			No zeros			Trimmed		
	Rate ratio	95% CI	*P*-value	Rate Ratio	95% CI	*P*-value	Rate Ratio	95% CI	*P*-value
50 m									
Both	0.63	(0.40, 0.998)	0.049	0.64	(0.40, 1.03)	0.063	0.65	(0.40, 1.06)	0.086
Bars only	0.60	(0.37, 0.97)	0.039	0.56	(0.34, 0.92)	0.023	0.67	(0.40, 1.12)	0.126
Restaurants only	0.78	(0.24, 2.47)	0.668	0.82	(0.26, 2.58)	0.728	0.68	(0.18, 2.59)	0.570
100 m									
Both	0.92	(0.64, 1.30)	0.622	0.91	(0.63, 1.32)	0.626	0.90	(0.62, 1.31)	0.586
Bars only	0.83	(0.57, 1.21)	0.335	0.81	(0.55, 1.19)	0.272	0.89	(0.60, 1.32)	0.557
Restaurants only	1.46	(0.70, 3.07)	0.312	1.39	(0.63, 3.07)	0.411	1.29	(0.51, 3.25)	0.595

*Adjusted for the amount of revenue from alcohol of each unit, the gun-free status of the unit, number of gun-free and gun-allowing establishments within 200 m from the unit, total revenue from alcohol sold within 200 m of the unit, population density, percent of racialized non-White population, percent poverty, median income, and percent of population age 18–35

The adjusted rate ratios for shootings within a 100-m radius are also given in Table 2. There were no statistically significant associations using this larger buffer. The analyses that excluded January through March 10, 2021, also resulted in near identical results to the primary findings.

## Discussion

Establishments that were gun-prohibiting in Texas in 2021 and 2022 had fewer shootings in and around their premises than establishments that were gun-allowing, after adjusting for confounders, including alcohol sales revenue. This association was significant when we examined bars only but not restaurants. This is perhaps due to the ways in which these establishments are utilized by customers and the nature of interactions. For example, restaurants often close earlier than bars and are more family-friendly. However, the lower number of gun-prohibiting restaurants in our study may have also contributed to the lack of significance, as this reduced our statistical power to detect meaningful differences for this group. As a whole, these findings were consistent across sensitivity analyses and align with established research on the behavioral and spatial dynamics of alcohol-serving venues and violence [33, 34]. 

The difference in shootings was statistically significant when the buffer size around the bars and restaurants was 50 m but not 100 m. This may be because areas further from the bar or restaurant are less influenced by the gun-free zone status of that venue, and that the 100 m buffers introduce noise to our estimates. A greater proportion of individuals 50 to 100 m from the establishment may not intend to enter, nor be aware of the existence of the policy.

These results are in line with the limited research on the effect of gun-free zones. A study on the effect of gun-free school zones and crimes committed with a firearm in Saint Louis, MO found that gun-free zones in their entirety (1000 feet from the school) were not associated with a difference in crimes committed with a firearm compared to other areas immediately around the gun-free school zone; however, there did seem to be a protective association immediately around the premises of the school [10]. Another study of gun-free zones on college and university campuses, however, did not find an association with rates of violence [35]. Additionally, a nationwide case–control study on the association between gun-free zones and active shootings found a protective association with gun-free zones on the occurrence of active shootings [36]. These findings also concur with previous research indicating that increased firearm availability correlates with a rise in gun-related injuries and deaths [37, 38].

### Limitations

We were unable to crosswalk all of the restaurants and bars across data sources. We spoke to the administrators of both datasets to improve linking, but record keeping was inconsistent. We do not believe that misspelled names, slight variations in business names across datasets, or typos would be associated with either the exposure (gun-free status) or the outcome (shootings), and therefore these omissions, to the extent that they were the cause of failed linkages, should not have biased our results. Nevertheless, to understand how these omissions might affect our findings, we conducted a comparative analysis of characteristics between the bars and restaurants included in our study and those that were not. This comparison showed that in 2022, the establishments that we were able to link on average had higher alcohol sales revenue ($535,992 versus $358,256 for excluded establishments). Moreover, a smaller proportion of these linked establishments prohibited firearms (8.7% as opposed to 14.7% among the excluded), and a slightly higher percentage were classified as bars (22.9% against 20.7%). To address potential biases introduced by these discrepancies, we incorporated these variables (alcohol sales revenue, firearm prohibition status, and establishment type) into our analysis. This approach aimed to mitigate the impact of the observed limitations on our study’s conclusions. Our ability to generalize to all establishments selling alcohol in Texas is hindered by these omissions. However, we have no reason to believe a similar association would not hold among the excluded bars and restaurants.

Another limitation worth noting is that our measure of alcohol consumption was based on the total revenue for alcohol sold. Places that sell more high-end liquor likely sell a lower volume of alcohol per dollar of revenue than places with lower-quality products, resulting in some measurement error. Additionally, while we did obtain time-varying data on the volume of alcohol sold by each establishment, our dataset did not include a similar time-varying measure for red sign designation (indicating gun-free status) for the years 2021 and 2022. The dataset, provided by the TABC, covered both years but recorded the red sign status in only one column. This limitation may have introduced non-differential misclassification of our exposure variable.

Texas was chosen as the location for this cross-sectional study due to the availability of data on gun-prohibited status and alcohol revenue by establishment and due to their consistently applied criteria for a bar or restaurant being designated as gun-prohibited. The results of this study may only be generalizable to Texas. We restricted the analysis to 2021 and 2022, as 2020 consisted of abnormalities presented by the pandemic. For example, any establishment that received more than 50% of their revenue from alcohol in Texas was shut down during the height of the COVID-19 pandemic, and other establishments were limited to 50% occupancy, which would make 2020 data untenable for this study [39]. Complicating matters further, many establishments that sold above this threshold temporarily converted to selling below the 50% mark, blurring the distinction between gun-free and gun-allowing establishments [40]. By 2021, Texas had mostly returned to business as usual [31], as indicated by the similar median alcohol revenue between 2021 ($251,572) and 2022 ($295,305). Future studies should explore the effect of gun-free zones in places that sell alcohol in different time periods and jurisdictions to establish consistency.

Finally, there is the possibility of omitted variable bias. For example, it is also possible that areas with high rates of gun violence are also areas where people consume more alcohol relative to other non-alcoholic goods at bars and restaurants, so the difference we see may not be explained only by the policy, but also this difference in behavior.

## Conclusion

After adjusting for confounders, including alcohol revenue, establishments that sold alcohol in Texas in 2022 that were gun-prohibiting had fewer shootings on and near their premises on average than establishments that were gun-allowing. This association was significant in bars but not restaurants, suggesting other features that differ between bars and restaurants may serve as important moderators of the effect. However, we cannot rule out the importance of such laws in restaurants as we may have been underpowered to detect differences in this smaller sample. Our study results suggest that gun-free zones may be effective at preventing firearm violence, although future studies using study designs that can support causal inference are needed to provide stronger support for such a conclusion.

## Data Availability

Data is available upon request.

## Appendix

Table

**Table 3:** Associations between all of the covariates and the outcome

Variable	Estimate	95% CI	*P*-value
Gun-free	0.630	(0.401, 0.998)	0.049
Revenue	1.000	(1.000, 1.000)	0.002
Percentage of those 18 to 35	1.012	(1.002, 1.02)	0.011
Median household income (in thousands)	1.003	(0.997, 1.009)	0.345
Percent of population racialized as non-White	1.018	(1.011, 1.025)	< 0.001
Percent in poverty	1.010	(0.996, 1.024)	0.171
Population density	1.000	(1.000, 1.000)	0.047
Nearby alcohol sales	1.000	(1.000, 1.000)	0.559
# of nearby non-gun-free establishments (200 m)	1.030	(1.004, 1.056)	0.023
# of nearby gun-free establishments (200 m)	1.071	(0.967, 1.186)	0.185
Establishment type (bar = 1, restaurant = 0)	3.175	(2.240, 4.501)	< 0.001
Year (2022 = 1, 2021 = 0)	0.928	(0.734, 1.174)	0.535

^3^
